# Development of a prediction model for hypotension after induction of anesthesia using machine learning

**DOI:** 10.1371/journal.pone.0231172

**Published:** 2020-04-16

**Authors:** Ah Reum Kang, Jihyun Lee, Woohyun Jung, Misoon Lee, Sun Young Park, Jiyoung Woo, Sang Hyun Kim

**Affiliations:** 1 SCH Media Labs, Soonchunhyang University, Asan, Chungnam, South Korea; 2 Department of Anesthesiology and Pain Medicine, Soonchunhyang University Bucheon Hospital, Soonchunhyang University College of Medicine, Bucheon, Gyeonggi, South Korea; 3 Department of Anesthesiology and Pain Medicine, Soonchunhyang University Seoul Hospital, Soonchunhyang University College of Medicine, Seoul, South Korea; Cleveland Clinic, UNITED STATES

## Abstract

Arterial hypotension during the early phase of anesthesia can lead to adverse outcomes such as a prolonged postoperative stay or even death. Predicting hypotension during anesthesia induction is complicated by its diverse causes. We investigated the feasibility of developing a machine-learning model to predict postinduction hypotension. Naïve Bayes, logistic regression, random forest, and artificial neural network models were trained to predict postinduction hypotension, occurring between tracheal intubation and incision, using data for the period from between the start of anesthesia induction and immediately before tracheal intubation obtained from an anesthesia monitor, a drug administration infusion pump, an anesthesia machine, and from patients’ demographics, together with preexisting disease information from electronic health records. Among 222 patients, 126 developed postinduction hypotension. The random-forest model showed the best performance, with an area under the receiver operating characteristic curve of 0.842 (95% confidence interval [CI]: 0.736-0.948). This was higher than that for the Naïve Bayes (0.778; 95% CI: 0.65-0.898), logistic regression (0.756; 95% CI: 0.630-0.881), and artificial-neural-network (0.760; 95% CI: 0.640-0.880) models. The most important features affecting the accuracy of machine-learning prediction were a patient’s lowest systolic blood pressure, lowest mean blood pressure, and mean systolic blood pressure before tracheal intubation. We found that machine-learning models using data obtained from various anesthesia machines between the start of anesthesia induction and immediately before tracheal intubation can predict hypotension occurring during the period between tracheal intubation and incision.

## Introduction

Arterial hypotension during surgery occurs frequently and is associated with adverse patient outcomes [1, 2]. Hypotension during the early phase of anesthesia, so-called postinduction hypotension (PIH), is related to multiple causative mechanisms, such as the patient’s age, preinduction systolic blood pressure (SBP), and emergency surgery [3]. In addition to these factors, comorbidity, preoperative use of medications, and anesthesia techniques, including the type and dose of anesthetic agent administered, also contribute to the development of PIH. Given these complex causes, prediction of hypotension during anesthesia induction remains difficult. If PIH could be accurately predicted, anesthesiologists would thereby be able to determine appropriate management proactively, thus preventing the negative outcomes associated with hypotension. These days, modern anesthesia data have been expanded to include high-resolution time-synchronized physiological and pharmacological data from multiple anesthesia devices [4]. This adds a large amount of anesthesia-related data to traditional electronic health records (EHRs); however, in a busy operating room environment it is not easy for an anesthesiologist to analyze these data in real time to predict PIH occurrence. Considering this, machine learning can be used as an alternative to assist the anesthesiologist in predicting PIH using such data. In the field of anesthesiology, various machine-learning models have been introduced to predict postoperative in-hospital mortality [5], hypotension [6], and PIH [7], showing prediction performance similar to, or better than, traditional modeling. We investigated the feasibility of developing a machine-learning model to predict PIH by adding intraoperative vital signs and anesthetic drug administration data obtained through high-resolution time-synchronized intraoperative data-mining techniques to EHR data.

## Materials and methods

### Patient population

Adult patients (age > = 18 years) who underwent laparoscopic cholecystectomy under general anesthesia at Soonchunhyang University Bucheon Hospital, Bucheon City, Republic of Korea, between October 29, 2018, and May 5, 2019, were included in this retrospective study. EHR and anesthesia data were retrieved from our department’s web database. Briefly, intraoperative vital signs and pharmacological data from multiple anesthesia drug-delivery and monitoring devices were recorded in a time-synchronized fashion using a Vital Recorder [8]. Together with these data, EHR data were also stored in our departmental database. The construction of the database was approved by our institutional review board (approval No. 2018-06-012). Additional approval from our institutional review board was obtained for this specific study (approval No. 2019-07-012).

### Primary outcome

We divided the PIH into two phases (early and late PIH). In this study, early PIH refers to hypotension occurring during the early phase of anesthesia induction, which is from the start of anesthesia to tracheal intubation, and late PIH is defined as hypotension occurring during the late phase of anesthesia induction, which is from tracheal intubation to incision. The primary outcome of the current study was the prediction of late PIH by machine-learning data obtained in the early phase of anesthesia induction. Hypotension was defined as SBP < 90 mmHg or a mean blood pressure (MBP) < 65 mmHg.

### Anesthesia

When the patient arrived in the operating room, routine monitoring was initiated, including electrocardiography, pulse oximetry, intermittent noninvasive blood pressure measurements, and bispectral index scoring. General anesthesia was induced and maintained with total intravenous anesthesia (TIVA) using propofol and remifentanil via a target-controlled infusion (TCI) pump (Orchestra Base Primea with module DPS; Fresenius Kabi AG, Germany) that contained a microprocessor programmed with pharmacokinetic models for each drug. The concentrations of propofol and remifentanil at the effect site (i.e., brain) were typically set at 3–6 *μ*g/mL and 2–6 ng/mL, respectively. After loss of consciousness, 0.6–1 mg of rocuronium was given intravenously to facilitate tracheal intubation. Thereafter, anesthesia was maintained with the target propofol and remifentanil concentrations titrated by the attending anesthesiologist’s clinical judgement based on the patient’s clinical signs and bispectral index score.

### Data collection

Demographic data (age, sex, height, weight, body mass index (BMI), American Society of Anesthesiologists (ASA) physical status grade, and underlying disease type) and data recorded from the Vital Recorder (vital signs, parameters related to mechanical ventilation, pharmacologic data such as propofol, remifentanil, and vasoactive drug administration) from anesthesia induction to incision were retrieved from our database. The timing of anesthesia induction was defined as the initial administration of propofol via a TCI pump. Table 1 summarizes the data collected from the EHR and Vital Recorder. Baseline blood pressure and heart rate were recorded when the first blood pressure and heart rate measurements were obtained in the operating room.

**Table 1:** Data description.

Data source	Categories	Features
Electronic Health Record	Demographic data	Age
		Sex
		Height
		Weight
		Body mass index
		ASA classification
	Comorbidities	Cardiovascular disease
		Respiratory disease
		Gastrointestinal disease
		Renal disease
		Endocrine disease
		Neurologic disease
Vital Recorder	Anesthetic drug^†^	Volume
		Plasma concentration
		Effect-site concentration
		Target concentration
	Vasoactive drug administration^†^	Vasopressor
		Vasodilator
	Noninvasive blood pressure^†^	Systolic
		Mean
		Diastolic
	Baseline blood pressure^‡^	Systolic
		Mean
		Diastolic
	Heart rate^†^	Heart rate
	Mechanical ventilation data^†^	Tidal volume
		Minute ventilation
		Respiratory rate
		Max positive airway pressure
	Hypotension^†^	Frequency
		Duration
		Average duration

The table notes the demographic information of the patient recorded in the electronic health record, derived features of the biomedical signal data, anesthetic drug derivative, hypotensive information, disease information in the pre-anesthetic history field, and drug information administered during anesthesia.

^†^These features were observed from anesthetic induction to tracheal intubation.

^‡^Baseline blood pressure was defined as the initial blood pressure in the operating room.

### Data analysis

#### Data collection and preprocessing

In addition to demographic data, vital signs, propofol, and remifentanil data collected by the Vital Recorder from different devices were collected. The intervals of the recording differed depending on the properties of the vital sign being measured. For example, blood pressure measured noninvasively was recorded once a minute, whereas the target-controlled infuser was recorded irregularly (e.g., at intervals of 1, 3, or 5s). We unified the bio-signal information record interval to 3 s for machine learning and replaced it with the last value if there was a gap in the data due to the time difference. Each record of a vital sign recorded in 3-s increments was labeled as 1 for hypotension according to the criteria above.

#### Data exploration

The physiological and pharmacological data were divided into two periods: (1) early-phase anesthesia induction, i.e., data from anesthetic induction to tracheal intubation, and (2) late-phase anesthesia induction, i.e., data from tracheal intubation to incision. The period was classified as hypotension if there was more than one hypotension label between tracheal intubation and incision or normal if there was no hypotension label in the same period.

#### Data from anesthetic induction to tracheal intubation

The frequency and duration of early PIH (hypotension occurring between anesthetic induction and tracheal intubation) were used as input features for late PIH prediction, corresponding to hypotension occurring between tracheal intubation and incision.

#### Data from tracheal intubation to incision

The hypotension information after tracheal intubation was used as an output class for machine learning (late PIH). The occurrence of hypotension was assigned if there was more than one hypotension label between tracheal intubation and incision. We also considered hypotension to have occurred when drugs that increase blood pressure were administered between tracheal intubation and incision, as these drugs indicate a response to signs of hypotension. This resulted in two patients being switched from the normal class to the hypotension class.

## Statistical analysis

### Feature

In this study, we used a total of 89 features. To avoid issues associated with dimensionality, we performed feature selection using the caret R package. Table 2 shows the features selected by feature selection.

**Table 2:** Features.

All features (89)	Feature set A (42)	Feature set B (20)	Feature set C (23)
Age	•	•	•
Sex	•		
Height			
Weight			
Body mass index	•		
ASA classification			
Comorbidities			
Cardiovascular disease			
Hypertension	•		
Atrial fibrillation			
Coronary artery disease	•		
Angina pectoris	•		
Congestive heart failure	•		
Respiratory disease			
Asthma	•		
Chronic obstructive pulmonary disease	•		
Gastrointestinal disease			
Hepatitis	•		
Liver cirrhosis	•		
Viral carrier	•		
Hepatitis B viral infection			
Renal disease			
Chronic kidney injury	•		
End-stage renal disease	•		
Endocrine disease			
Diabetes mellitus	•		
HbA1c			
Thyroid disease	•		
Neurologic disease			
Cerebrovascular disease	•		
Cerebral aneurysm	•		
Baseline blood pressure			
Systolic			•
Mean			•
Diastolic		•	•
Noninvasive blood pressure^†^			
Systolic min		•	•
Systolic max			•
Systolic mean		•	•
Systolic sd		•	•
Mean min		•	•
Mean max			•
Mean mean		•	•
Mean sd	•	•	
Diastolic min			•
Diastolic max			
Diastolic mean		•	•
Diastolic sd			
Heart rate^†^			
min	•		
max	•	•	
mean		•	
Anesthetic drug^†^			
Volume			
Propofol min			
Propofol max	•	•	•
Propofol mean		•	•
Remifentanil min	•		
Remifentanil max		•	•
Remifentanil mean	•	•	•
Plasma concentration			
Propofol min			
Propofol max	•	•	•
Propofol mean			
Remifentanil min			
Remifentanil max			
Remifentanil mean			
Effect-site concentration			
Propofol min			
Propofol max			
Propofol mean			
Remifentanil min	•		
Remifentanil max			
Remifentanil mean			•
Target concentration			
Propofol min	•		
Propofol max			
Propofol mean			
Remifentanil min	•		
Remifentanil max			
Remifentanil mean	•	•	
Vasoactive drug administration^†^			
Ephedrine			
Ephedrine volume	•		
Phenylephrine	•		
Phenylephrine volume			
Esmolol	•		
Esmolol volume			
Labetalol	•		
Labetalol volume			
Mechanical ventilation data^†^			
Tidal volume min	•		
Tidal volume max	•	•	
Tidal volume mean		•	•
Minute ventilation min			
Minute ventilation max	•		
Minute ventilation mean			
Respiratory rate min	•		
Respiratory rate max	•		
Respiratory rate mean		•	
Max positive airway pressure min	•		
Max positive airway pressure max	•		
Max positive airway pressure mean	•		
Hypotension^†^			
Frequency			•
Duration			•
Average duration	•		•

Initially, 100 features were selected; however, no patients had 11 comorbidities: myocardial infarction, valve disease, hepatitis C viral infection, fatty liver, alcoholic liver disease, autoimmune liver disease, acute kidney injury, myasthenia gravis, morbid obesity, epilepsy, or dementia. Thus, these features were excluded.

^†^Features observed between anesthesia induction and tracheal intubation.

#### Feature selection

We extracted 89 features from EHR and Vital Recorder data to predict late PIH using machine learning. The number of pieces of information for each sample is the dimensionality. Large dimensionality makes model training difficult and requires more data [9]. The more information we know, the more possibilities we can utilize, but large dimensionality is not always good. The performance of a model may be high without unnecessary variables, but decline as unnecessary variables are added. As dimensionality increases with additional training data, the performance may drop sharply. In addition, if the number of variables exceeds the training data, the model may not explain new data. When features are added, the model becomes more complex and is more likely to overfit. Therefore, it is better to select only the most useful features. The training data used in this study comprised 75% (166 cases) of the total data. If we had used all 89 extracted features, there would have been a high risk of dimensionality complications. To prevent over-fitting and improve performance, we applied three feature selection strategies.

First, redundant features were removed. The data included correlated attributes. Many methods perform better when highly correlated attributes are removed. In this study, we removed attributes with an absolute correlation coefficient of 0.5 or greater. Forty-two features were selected and defined as Feature set A.

Second, features were ranked by their importance. The importance of a feature can be estimated from the data after the model is created. Some methods, such as decision trees, have built-in mechanisms to report variable importance. For other algorithms, the importance can be estimated using the receiver operating characteristic (ROC) curve analysis performed for each feature. Twenty features were selected and defined as Feature set B.

Last, specific features were selected using the recursive feature elimination (RFE) method, a popular automatic method for feature selection provided by the caret R package. This is a greedy optimization algorithm that is used to find the best performance variable. RFE is a wrapper method that uses a subset of variables to learn the model, allowing the addition or subtraction of features from previous models based on inference; it continues to generate the model and keeps the best- or worst-performing models. It considers the next model until all of the variables are gone, and then ranks the variables according to their removal order. Using the RFE method, 20 features were selected and defined as Feature set C. The experimental results for each feature are summarized in the next section.

### Machine learning model

We developed Naïve Bayes, logistic regression, random forest, and artificial neural network (ANN) models for predicting late PIH. Naïve Bayes is a probabilistic classifier applying the Bayesian theorem that assumes independence between properties [10]. Logistic regression is a probabilistic model that uses the relationship between dependent and independent variables as a concrete function for prediction models [11]. Random forest randomly samples training data to create a large number of decision trees and then collects the results of the decision trees to derive the final result by majority vote [12]. The decision tree predicts the value of the target variable according to several input variables. Random forest has high accuracy because it generates a large number of these decision trees, collectively learns them, and derives a majority result. It is also simple and fast, and it can handle large data sets and many input variables. ANN mimics the brain’s information processing system, which involves complex neuron connections and complex computations [13]. ANN derives a new value through a predetermined function process when various information is input. The advantage of this algorithm is that any estimation function can be approximated by reasonably complex neural networks with high prediction accuracy. We performed repeated k-fold cross-validation to gurantee unbiased performance. K-fold cross validation method is a statistical skill to measure the performance of the model on new data after splitting the data into k folds. A fold is tested as new data for the model built from remining k-1 folds, and this process is repeated while all folds are tested once. K-fold validation has randomness in sample selection in forming a fold. When samples are homogeneous, the randomness would not cause biased performance on a specific fold split.

However, when samples are heterogeneous, the algorithm performance could change depending which samples are split into which fold. The repeated k-fold validation complements this weakness by repeating the step splitting samples into folds n-times. Bio-medical data, especially our bio-sensor data is diverse depending on patients, so we repeated four-fold cross-validation 1000 times to generate stable performance.

### Performance evaluation

The performance of the learning models is summarized using the area under the ROC (AUC), accuracy, precision, and recall [14]. The evaluation of the model was based on whether the answer given by the model matched the actual answer. Eq 1 describes the indices as True Positive (TP), True Negative (TN), False Positive (FP), and False Negative (FN).

$$\begin{array}{c} \begin{array}{cc} precision & =\frac{TP}{TP+FP}\\ recall & =\frac{TP}{TP+FN}\\ accuracy & =\frac{TP+TN}{TP+FN+FP+TN} \end{array} \end{array}$$ (1)

### Determining the importance of explanatory features

The explanatory power of a model lies in its ability to identify the relative importance of the explanatory features that affect the target output. To measure the importance of explanatory variables in random forest, two importance indicators are used: the mean decrease in accuracy and the mean decrease in Gini. The mean decrease in accuracy is a measure of the importance of a variable based on its accuracy [15] and is defined as the average difference in accuracy that occurs when a variable is removed and the model is rebuilt. If the accuracy is greatly reduced by eliminating a variable, the variable has a significant effect on improving classification accuracy. The mean decrease in Gini is a measure of the decrease in the impurity of the selected variables as each tree in the random forest extends its branches and uses the average value from the entire tree. A higher mean decrease in Gini value for a particular variable means that sorting individuals using that variable helps to group categories in a way that reduces impurity. Therefore, both of the indicators that measure the importance of variables in random forest have high importance if the values are large. A Shapiro–Wilk test for normality was conducted, and t-tests or Wilcox tests for continuous data were performed based on the outcome. Categorical variables were evaluated using a chi-squared or Fisher’s exact test.

## Results

The data from a total of 222 patients were analyzed. Among these, 126 patients developed late PIH. Patients who developed late PIH tended to be significantly older and have a lower baseline blood pressure compared with patients who did not. Effect site concentration of propofol and remifentanil were not significantly different. The characteristics of patients are specified in Table 3.

**Table 3:** Patient characteristics.

Characteristic	All patients (n = 222)	Hypotension (n = 126)	No hypotension (n = 96)	p
Age—*yr*	53 (14)	55.7 (14.2)	49.5 (13)	0.001***
Sex (male)	104 (46.9%)	54 (42.9%)	50 (52.1%)	0.219
Height—*cm*	162.3 (9.3)	161.6 (9.7)	163.2 (8.7)	0.199
Weight—*kg*	65.2 (57, 73.8)	63.2 (55.1, 72.9)	66.9 (58.9, 77.2)	0.023*
BMI—*kg*/*m*^2^	24.7 (22.5, 27.3)	24.4 (22.3, 26.8)	25.1 (23.1, 27.4)	0.05*
ASA classification—no.				0.509
1	81 (36.5%)	42 (33.3%)	39 (40.6%)	
2	117 (52.7%)	69 (54.8%)	48 (50%)	
3	24 (10.8%)	15 (11.9%)	9 (9.4%)	
Comorbidities—no.				
Cardiovascular disease				
Hypertension	71 (32%)	42 (33.3%)	29 (30.2%)	0.727
Atrial fibrillation	3 (1.4%)	3 (2.4%)	0 (0%)	0.35
Coronary artery disease	4 (1.8%)	3 (2.4%)	1 (1%)	0.815
Angina pectoris	5 (2.3%)	2 (1.6%)	3 (3.1%)	0.758
Congestive heart failure	1 (0.5%)	0 (0%)	1 (1%)	0.891
Respiratory disease				
Asthma	16 (7.2%)	13 (10.3%)	3 (3.1%)	0.073
Chronic obstructive pulmonary disease	5 (2.3%)	2 (1.6%)	3 (3.1%)	0.758
Gastrointestinal disease				
Hepatitis	1 (0.5%)	1 (0.8%)	0 (0%)	>0.99
Liver cirrhosis	4 (1.8%)	3 (2.4%)	1 (1%)	0.815
Viral carrier	7 (3.2%)	4 (3.2%)	3 (3.1%)	>0.99
Hepatitis B viral infection	11 (5%)	6 (4.8%)	5 (5.2%)	>0.99
Renal disease				
Chronic kidney injury				0.453
2	1 (0.5%)	0 (0%)	1 (1%)	
3	4 (1.8%)	3 (2.4%)	1 (1%)	
4	1 (0.5%)	1 (0.8%)	0 (0%)	
End-stage renal disease	1 (0.4%)	1 (0.8%)	0 (0%)	>0.99
Endocrine disease				
Diabetes mellitus	45 (20.3%)	25 (19.8%)	20 (20.8%)	0.989
Thyroid disease	14	8	6	0.498
Neurologic disease				
Cerebrovascular disease	9 (4.1%)	6 (4.8%)	3 (3.1%)	0.788
Cerebral aneurysm	1 (0.5%)	0 (0%)	1 (1%)	0.891
Baseline blood pressure—*mmHg*				
Systolic	141 (127, 160)	138 (123, 154.8)	145.5 (133, 165)	0.001***
Mean	104 (92, 113)	98 (90, 110)	108 (98.8, 118)	<0.001***
Diastolic	80.4 ±10.95	77.8 ±11	84 ±9.9	<0.001***
Noninvasive blood pressure^†^—*mmHg*				
Systolic	118 (107.7, 128.3)	111 (102.9, 122.5)	123 (116, 132.9)	<0.001***
Mean	87.3 (80.2, 95.3)	82.7 (76.7, 91.4)	91.1 (86.5, 99)	<0.001***
Diastolic	70 ±9.5	75 ±11.23	74.6 ±10.7	<0.001***
Heart rate^†^—/*min*	75 ±11	75 ±11.2	74 ±10.7	0.767
Anesthetic drug^†^				
Effect-site concentration				
Propofol (*mcg*/*ml*)	4.2 (3.8, 4.6)	4.2 (3.8, 4.5)	4.3 (3.9, 4.7)	0.208
Remifentanil (*ng*/*ml*)	1.6 (1, 1.9)	1.5 (1, 1.8)	1.6 (1.2, 1.9)	0.251
Vasoactive drug administration^†^—no.				
Ephedrine	6 (2.7%)	6 (4.8%)	0 (0%)	0.08
Esmolol	4 (1.8%)	2 (1.6%)	2 (2.1%)	>0.99
Labetalol	1 (0.5%)	0 (0%)	1 (1%)	0.891
Mechanical ventilation data^†^				
Tidal volume—*ml*	271.7 (223.6, 321.9)	268 (217.4, 320.7)	275.4 (232, 346.3)	0.332
Minute ventilation—*L*/*min*	3.3 (2.6, 4.2)	3.3 (2.6, 4.1)	3.3 (2.7, 4.5)	0.543
Respiratory rate—/*min*	11.7 (9.9, 14.1)	11.9 (9.8, 14.1)	11.6 (10.2, 14.2)	0.762
Max positive airway pressure—*cmH*2*O*	14.8 ±3.3	14.6 ±3.3	15 ±3.3	0.426

Data are expressed mean (standard deviation, SD) or median (interquartile range, IQR) values according to normality test results. For continuous variables, a t-test or Wilcox test was performed as appropriate. For categorical variables, a chi-squared test or Fisher’s exact test was performed as appropriate.

^†^These features were observed from anesthetic induction to tracheal intubation.

* *p* < 0.05,

** *p* < 0.01, and

*** *p* < 0.001.

### Late PIH: Hypotension after tracheal intubation

**Fig 1:**
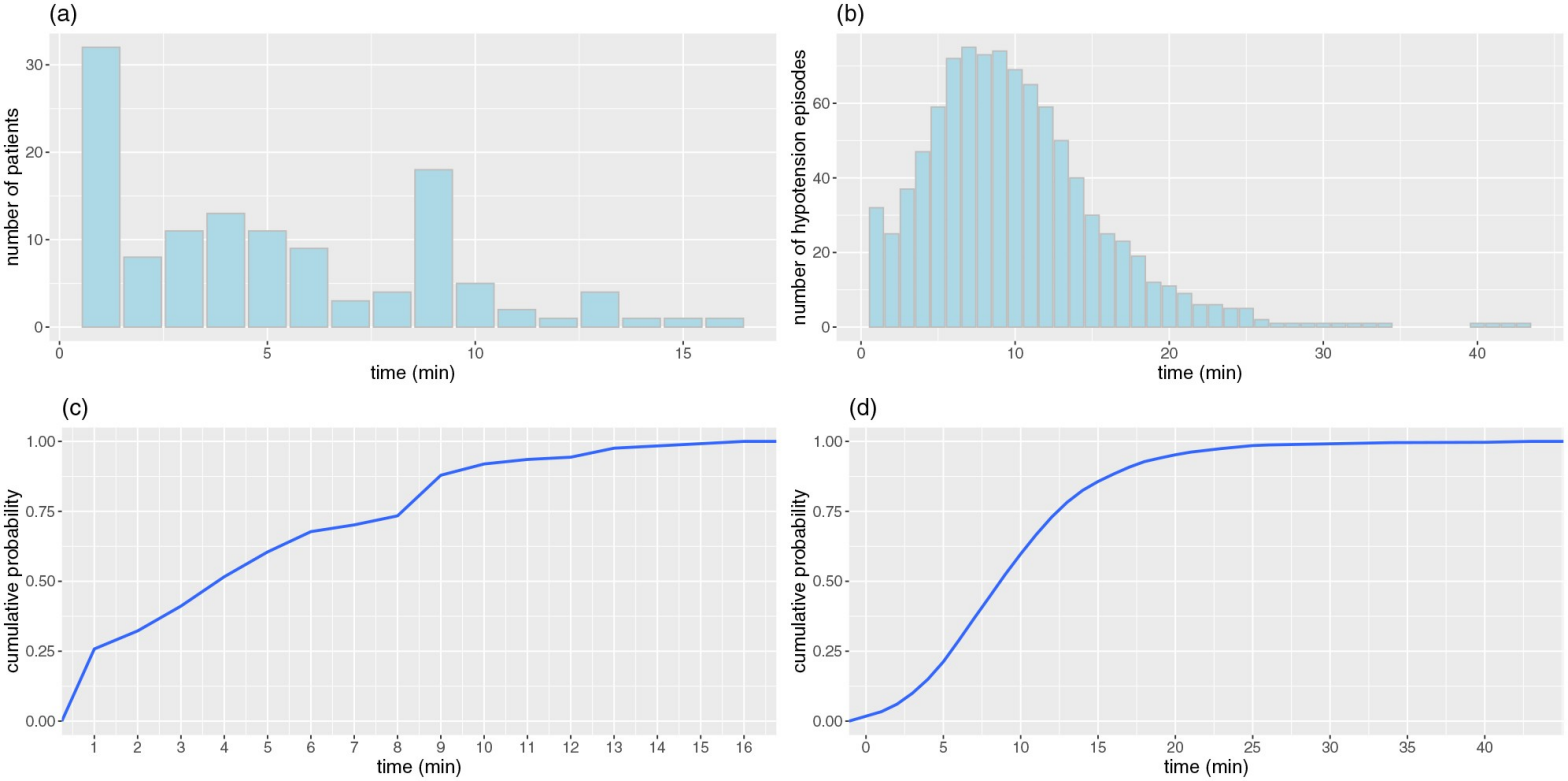
Distribution of hypotension after tracheal intubation. (a) the distribution of the elapsed time from tracheal intubation to first hypotension event in terms of patients. (b) the distribution of the elapsed time from tracheal intubation in terms of hypotension episodes. (c) the cumulative distribution of the elapsed time from tracheal intubation to first hypotension event in terms of patients. (d) the cumulative distribution of the elapsed time from tracheal intubation in terms of hypotension episodes.

Fig 1 plots late PIH from two perspectives. Fig 1(a) and 1(c) present the distribution of patients showing the first hypotension episode over time. In Fig 1(a), 32 of the 126 patients with hypotension experienced their first hypotension within one minute immediately after tracheal intubation. In Fig 1(c), within 4 minutes after tracheal intubation, 50% of 126 patients experienced their first hypotension. Fig 1(b) and 1(d) show the number of hypotension episodes after tracheal intubation over time. The number of hypotension episodes peaked at around 10 min and then decreased. Fig 1(d) indicates that 70% of the hypotension episodes occurred within 12 min. The slope of Fig 1(d) gradually increased from 1 min to 3 min, before rising sharply from 5 min to 12 min. After 12 min, the slope was more gradual. This means that many hypotension episodes occurred between 5 and 12 min. The average value was 10 min, and the median value was 9 min.

### Late PIH prediction performance among machine-learning models

**Fig 2:**
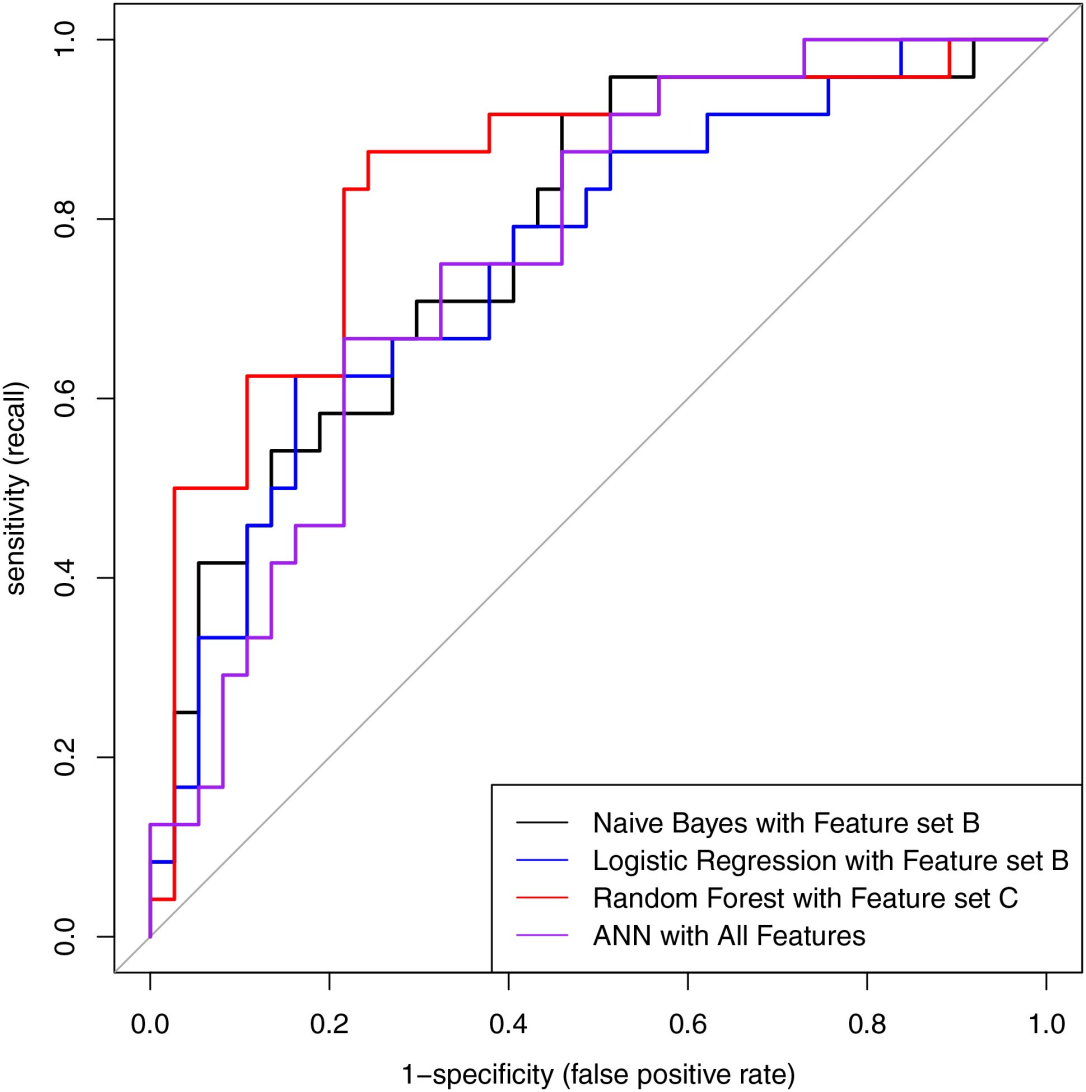
Receiver operating characteristic curve. Naïve Bayes, logistic regression, random forest, and ANN models are expressed as black, blue, red, and purple lines, respectively. Naïve Bayes with Feature set B (AUC, 77.82%), logistic regression with Feature set B (AUC, 75.56%), random forest with Feature set C (AUC, 84.23%), and ANN with All features (AUC, 76.01%). ANN, artificial neural network.

The performances of four machine-learning models in predicting late PIH are summarized in Table 4. Among the models, random forest performed best; specifically, the recall and AUC with feature set C were 83.65% and 84.23%, respectively (Fig 2).

**Table 4:** Performance of the four machine-learning models for PIH prediction.

		Naïve Bayes	Logistic regression	Random forest	ANN
**All features**	accuracy	55.74	62.01	76.28	70.7
	precision	80	70.25	77.99	74.51
	recall	13.33	60.79	81.28	74.5
	AUC	60.16	60.47	79.5	76.01
	95% CI	45.41–74.62	46.69–74.26	67.87–91.14	64.03–88
**Feature set A** (Remove redundant features)	accuracy	53.64	59.97	68.28	64.53
	precision	78.5	67.11	71.36	69.14
	recall	28.02	59.23	74.61	69.27
	AUC	67.23	66.78	71.85	70.72
	95% CI	53.19–81.27	52.74–80.81	58.58–85.1	56.98–84.47
**Feature set B** (Rank features by importance)	accuracy	70.2	79.16	78.8	70.61
	precision	71.54	79.95	79.5	72.92
	recall	79.71	85.01	84.58	77.64
	AUC	77.82	75.56	83.78	67.57
	95% CI	65.87–89.76	63.02–88.11	73.36–94.2	53.53–81.6
**Feature set C** (Recursive feature elimination)	accuracy	70.02	68.56	79.48	68.62
	precision	77.08	72.75	81.16	72.25
	recall	67.13	71.67	83.65	72.97
	AUC	77.25	73.42	84.23	72.3
	95% CI	65.13–89.38	60.57–86.27	73.63–94.84	59.5–85.09

ANN, artificial neural network; AUC, area under receiver operating characteristic curve. Table notes the precision and recall for the hypotension class.

**Fig 3:**
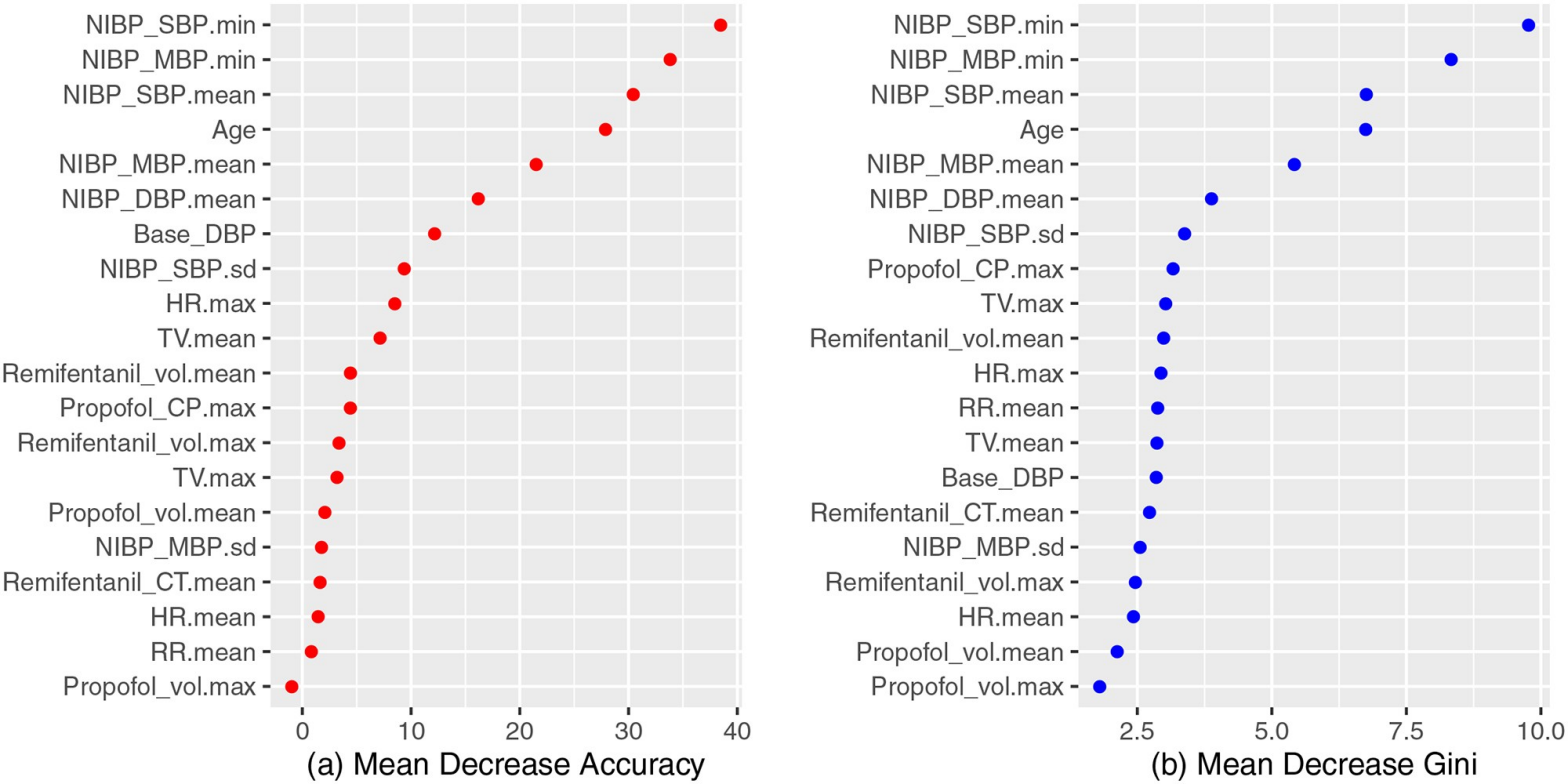
Feature importance plot from the random forest model. NIBP_SBP.min and NIBP_MBP.min were ranked as the first and second most important features based on the importance plot of the random forest model. NIBP, noninvasive blood pressure; SBP, systolic blood pressure; MBP, mean blood pressure; TV, tidal volume; CP, plasma concentration; HR, heart rate; DBP, diastolic blood pressure; RR, respiratory rate; CT, target concentration.

Fig 3 shows a feature-importance plot from the random forest model with two indicators. For the mean decrease in accuracy and the mean decrease in Gini, we see that NIBP SBP.min, NIBP MBP.min, NIBP SBP.mean, and age variables are located at the top of the graph (Fig 3).

## Discussion

In this study, we evaluated whether machine learning can be used to predict hypotension occurring after tracheal intubation. We trained our model using data obtained from the early phase of anesthesia induction. These data included not only EHR data but also early anesthesia induction data collected from various machines such as a general anesthesia monitor, drug infusion pump, mechanical ventilator, and anesthesia depth monitor. Our results show that: machine learning can predict late PIH with a variable range; among the four methods used, the random forest model showed the best performance (AUC = 0.84). Instead of using all 89 features, selected features (20 and 23 features) obtained using a feature-selection method provided the best performance. The three most important features affecting the accuracy of machine-learning (e.g., random forest) prediction were the patient’s lowest SBP, lowest MBP, and mean SBP before tracheal intubation. Among patients’ characteristics, patient age was an important factor in predicting late PIH.

PIH is not uncommon, occurring in about 20% of patients [1]. It is associated with a high prevalence of poor outcomes. Reich and colleagues reported that a prolonged postoperative stay and death were more common in patients with early PIH that occurred within 10 min after anesthesia induction [2]. In the current study, we investigated the occurrence of hypotension between tracheal intubation and incision. During this period, patients may experience a variety of conditions that make them more prone to hypotension. After intubation, anesthesiologists are busy working on several tasks, i.e., securing the tracheal tube, adjusting the anesthesia drug, inducing inspiratory oxygen flow and oxygen fraction, setting the tidal volume and respiratory rate, and simultaneously entering data into the patient’s anesthesia records; thus, it may be difficult for the anesthesiologist to concentrate fully on hemodynamic changes. Surprisingly, half of the patients in our study developed hypotension during this period, even though the hypotension threshold was set slightly higher (SBP of 90 mmHg or MBP of 65 mmHg) and anesthesia induction was provided solely by TIVA, which uses propofol and remifentanil, the main anesthesia drugs contributing to the development of hypotension. We assumed that this high incidence may largely have resulted from frequent blood pressure measurements (i.e., every 1 min) and might otherwise have gone undetected if the blood pressure measurements had been taken every 3 to 5 min. In this study, random forest showed the highest predictive performance among four machine-learning models. In general, random forest performed very well, with minimal parameter tuning, and did not require scale data; however, random forest did not work well with sparse data, such as text data. Each feature of the biosignal data was organically linked to the others. Thus, the Naïve Bayes classifier, which assumes independence between features, showed relatively poor performance. Logistic regression assumes only a linear relationship between a dependent variable and independent variables. There is a limit to increasing the accuracy. For example, ANN models tend to overfit their training data. Kendale and colleagues have shown that a gradient boosting machine algorithm had an AUC of 0.77 in predicting PIH within 10 min of the recorded induction time of general anesthesia [3].

When all 89 features were used, logistic regression and Naïve Bayes showed very poor prediction performance (AUC = 0.6). In the case of using Feature set A, the predictive ability varied, depending on the model. However, when Feature sets B and C were used, the predictive performance increased in all models except ANN. Among them, random forest showed the best performance. The main differences in Feature set A compared with sets B and C was that Feature set A included many of the existing-disease features (15 features) and excluded almost all of the blood pressure-related features. In contrast, Feature sets B and C had none of the existing-disease features, but most blood pressure-related features were included. In particular, Feature set C contained baseline blood-pressure and early PIH-related features that were not included in B. Thus, the results indicate that the use of existing disease as a feature in predicting late PIH does not improve prediction performance. However, the inclusion of blood-pressure-related factors improved the prediction accuracy.

Our analysis of important feature selection showed that lowest systolic pressure and MBP during the early phase of anesthesia induction were the two most important features in predicting late PIH. This implies that low blood pressure before tracheal intubation is associated with the development of late PIH. The high importance of these features seems reasonable to anesthesiologists, considering the general tendency of anesthesiologists to predict blood pressure a few minutes ahead by observing changes in blood pressure during the early phase of anesthesia induction. This implies that our model identified an intuitive component of predicting late PIH. Among other features, patient age was ranked as the fourth most important factor. This also makes sense, in that elderly patients are more vulnerable to developing hypotension than are relatively young patients. Apart from these clinical aspects, variables recently identified using traditional multivariate logistic regression models as significantly related to PIH include pre-induction SBP, age, and emergency surgery [3]. Considering that machine-learning predictions have taken the form of a black box and have not been able to provide convincing information to clinicians, the similarity between the high-priority features in our study and the variables resulting from traditional statistical approaches is meaningful, as it implies that machine-learning techniques can provide information that clinicians can understand. In addition, Kendale and colleagues, using a stochastic gradient boosting machine-learning algorithm, also reported that the first MBP and age were the two most important predictive features for PIH.

We assume that the relatively high prediction performance for late PIH in the current study comes mainly from including features generated during the early stage of anesthesia induction. In addition to EHR data, we can use features that may be directly related to the development of hypotension. For example, blood pressure, anesthesia drug, and mechanical ventilator data can be recorded in high resolution. Although these data can be obtained from EHRs (i.e., anesthesia records) retrospectively, the data may be incorrect, especially during the initial short period of anesthesia induction. Apart from blood pressure recording, intravenous anesthesia drug administration information cannot be recorded in anesthesia records to reflect real-time changes unless specialized tools are used. Mechanical ventilation data are not recorded routinely on anesthesia records and often missed unless they are recorded separately. However, these missing data are, in fact, clinically correlated with hypotension development. We were able to record and store blood-pressure and heart-rate data as well as anesthesia drug and mechanical ventilation information using a Vital Recorder. We found that propofol- and remifentanil-related features also played important roles in improving prediction performance. The incorporation of these features is expected to enhance hypotension prediction performance considerably.

## Limitations

There are several limitations to this study. We did not include post-tracheal intubation data to train the machine-learning model to predict late PIH. In fact, post-intubation drug control may or may not cause hypotension. However, we used data up to immediately before intubation on the premise that most anesthesiologists provide leveled anesthesia at incision after intubation. Our study was performed only on laparoscopic cholecystectomy using TIVA. The reason for this was as follows. First, the data from the infusion pump used in TIVA can be well recorded. Second, by limiting the operation type to the same operation, the patient group could consist of similar patients. Finally, the same procedure usually follows a common sequence from induction of anesthesia to incision. This helped to eliminate unexpected variables that might occur in other operations. For example, if we had included high-risk surgery that requires a procedure such as inserting a urinary catheter, radial artery catheter, nasogastric tube, or central venous catheter, this might have added additional factors that could have caused dimensionality complications. Thus, our study cannot be applied to other surgeries. The number of patients in this study was small, which could possibly lead to overfitting. However, of the 222 patients, 126 were in the hypotension group and 99 were in the normal group; thus, the sample appeared to be fairly balanced.

## Conclusion

We found that machine-learning models using data obtained from EHR and various anesthesia machines between the start of anesthesia induction and immediately before tracheal intubation can predict hypotension between tracheal intubation and incision. Random forest with features selected by RFE showed the best performance for identifying late PIH. Lowest SBP, lowest MBP, mean SBP before tracheal intubation, and patient age were important for the accuracy and node impurity of the random forest model.

## Supporting information

S1 Dataset This is our data used in this paper that can be used for replication studies. (CSV)
